# Correlation Functions Quantify Super-Resolution Images and Estimate Apparent Clustering Due to Over-Counting

**DOI:** 10.1371/journal.pone.0031457

**Published:** 2012-02-27

**Authors:** Sarah L. Veatch, Benjamin B. Machta, Sarah A. Shelby, Ethan N. Chiang, David A. Holowka, Barbara A. Baird

**Affiliations:** 1 Department of Biophysics, University of Michigan, Ann Arbor, Michigan, United States of America; 2 Department of Physics, Cornell University, Ithaca, New York, United States of America; 3 Department of Chemistry and Chemical Biology, Cornell University, Ithaca, New York, United States of America; Stanford, United States of America

## Abstract

We present an analytical method using correlation functions to quantify clustering in super-resolution fluorescence localization images and electron microscopy images of static surfaces in two dimensions. We use this method to quantify how over-counting of labeled molecules contributes to apparent self-clustering and to calculate the effective lateral resolution of an image. This treatment applies to distributions of proteins and lipids in cell membranes, where there is significant interest in using electron microscopy and super-resolution fluorescence localization techniques to probe membrane heterogeneity. When images are quantified using pair auto-correlation functions, the magnitude of apparent clustering arising from over-counting varies inversely with the surface density of labeled molecules and does not depend on the number of times an average molecule is counted. In contrast, we demonstrate that over-counting does not give rise to apparent co-clustering in double label experiments when pair cross-correlation functions are measured. We apply our analytical method to quantify the distribution of the IgE receptor (FcεRI) on the plasma membranes of chemically fixed RBL-2H3 mast cells from images acquired using stochastic optical reconstruction microscopy (STORM/dSTORM) and scanning electron microscopy (SEM). We find that apparent clustering of FcεRI-bound IgE is dominated by over-counting labels on individual complexes when IgE is directly conjugated to organic fluorophores. We verify this observation by measuring pair cross-correlation functions between two distinguishably labeled pools of IgE-FcεRI on the cell surface using both imaging methods. After correcting for over-counting, we observe weak but significant self-clustering of IgE-FcεRI in fluorescence localization measurements, and no residual self-clustering as detected with SEM. We also apply this method to quantify IgE-FcεRI redistribution after deliberate clustering by crosslinking with two distinct trivalent ligands of defined architectures, and we evaluate contributions from both over-counting of labels and redistribution of proteins.

## Introduction

Recent advances in super-resolution imaging have enabled imaging of cellular structures at close to molecular length scales using light microscopy [1], [2], [3], [4], [5]. In conventional fluorescence microscopy, the average distance between fluorescently labeled molecules is typically very small compared to the width of the point spread function (PSF) of the microscope (∼250 nm). In this limit, the fluorescence character of individual labeled molecules does not contribute significantly to the final image, since many individual labeled molecules are averaged within the PSF of the measurement. Super-resolution fluorescence imaging and localization techniques can improve lateral resolution by an order of magnitude. In this limit, the average distance between neighboring labeled molecules can be close to the resolution of the measurement, and the finite size of individual labeled molecules as well as the finite size of the measurement resolution can significantly impact the resulting images. For example, under-sampling of super-resolution images can lead to lower effective resolution by some measures, as discussed in previous work [6], [7], [8]. In this study, we explicitly assess how inadvertent over-sampling of individual labeled molecules can lead to the erroneous appearance of self-clustering. The situation can arise in both super-resolution localization images of fluorescently labeled proteins and in electron microscopic images of gold labeled proteins. When not considered explicitly, this apparent self-clustering could be incorrectly interpreted as self-clustering of labeled proteins. This is an important consideration since correctly determining the organization of membrane components is vital for deciphering how membrane organization is linked to cellular functions.

Over-counting of labels in nano-scale resolution imaging techniques is a common but under-appreciated problem. Over-counting can occur, for example, when target proteins are labeled with primary and secondary antibodies or when antibodies are conjugated to multiple fluorophores. It can also occur when the same fluorophore is counted two or more times because it cycles reversibly between activated and dark states. In all of these cases, over-counting can lead to the artifactual appearance of self-clustering over distances that correspond to the effective resolution of the measurement. In this study we first describe a method to quantify the distribution of labeled molecules in images, and we then develop a simple model to predict the magnitude of apparent clustering arising from over-counting. We show how this formalism applies to deliberate over-counting and thereby provides a useful measure of the effective average lateral resolution of a reconstructed super-resolution fluorescence localization image. We use this analytical approach to quantify high resolution images of the high affinity IgE receptor (FcεRI) on the surface of RBL-2H3 mast cells obtained using both stochastic optical reconstruction microscopy (STORM/dSTORM) and scanning electron microscopy (SEM). We also apply the method to an example of IgE-FcεRI complexes that are deliberately clustered on the cell surface by crosslinking with defined trivalent ligands. In this case, the observed clustering contains contributions from the redistributed proteins in addition to the inherent over-counting of multiple labels. Our approach can also be applied to other types of high resolution imaging methods, including transmission electron microscopy (TEM) and has recently been applied to quantify images obtained using photoactivated light microscopy (PALM/fPALM) [9].

## Results and Discussion

### Pair auto-correlation functions quantify over-counting

Pair correlation functions quantify organization in heterogeneous systems and are easily applied to super-resolution localization data. The pair auto-correlation function, 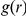, that reports the increased probability of finding a second localized signal a distance r away from a given localized signal, is efficiently calculated using Fast Fourier Transforms, and can account for complex boundary shapes without additional assumptions. Detailed methods used to calculate correlation functions are described in Materials and Methods, and a Matlab function to calculate 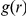 from images is supplied in File S1.

If an ensemble of molecules is distributed on a two dimensional surface with centers at positions 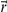 described by the density function 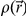 and an average density 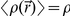, the associated pair auto-correlation function of molecular centers is:
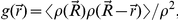
where the average is over all positions 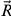 in the image. In this definition, 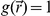 represents a random distribution. Often it can be assumed that 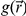 is symmetric to rotations, and it is averaged over angles to obtain 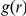. At 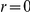, 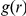 contains a delta function, 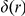, with magnitude of 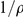. Correlation functions are plotted for 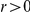, as 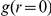 is a trivial contribution. However, if 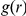 is calculated from an image obtained from a measurement with finite resolution in the presence of over-counting, the measured correlation function will contain a remnant of this delta function at nonzero radius:
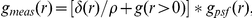
where 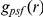 is the correlation function of the average PSF of the measurement, 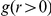 represents the correlation function for the distribution of labeled molecules, and 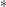 denotes a two dimensional convolution. The convolution acts to smear 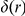 to finite radius. A detailed derivation of the above equation is included in Materials and Methods and a discussion of some important caveats are included later in this section.

**Figure 1:**
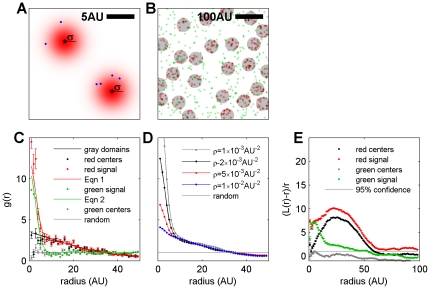
Simulated demonstration of apparent clustering arising from over-counting individual labeled molecules with a finite effective PSF. (A) Labeled molecules centered at black stars are convolved by a Gaussian PSF with half-width σ = 2 in arbitrary units (AU) (red areas). In this example, the red areas represent the finite resolution of the measurement that could arise from multiple factors, including finite localization precision in a super-resolution fluorescence localization measurement or the finite size of labeling antibodies in an SEM measurement. Blue points are examples of signals detected with probability given by the intensity of the red area. Here the over counting ratio (OCR) is 3, meaning each labeled molecule is counted on average 3 times. (B) Red labeled molecules are confined within gray circular domains with an average radius of 25 AU, while green labeled molecules are distributed at random. Both labeled molecules have an average surface density 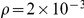 AU^−2^ and 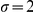 AU. (C) Correlation functions calculated from B for structures as indicated. Red (green) signals are sampled at random from red (green) PSF areas with OCR = 1, as described in A. g(r) for red centers and gray domains are equivalent within error, but g(r) for red signals shows additional clustering at short r, in agreement with Eqn 1. Green signals are also clustered at short r as described by Eqn 2, while g(r) for green centers is random within error. (D) Simulated g(r) for labeled red molecules partitioned into the gray domains as in B but with different average surface densities (ρ). Apparent clustering at short r decreases as ρ is increased, but long range correlations are unchanged, consistent with Eqn 1. (E) Modified Ripley's functions, (L(r)−r)/r, calculated from clustered red centers is slightly lower than but resembles functions calculated for red signals at large r. As expected, modified Ripley's functions for randomly distributed green centers do not show significant clustering over any radius. In contrast, functions calculated from green signals show significant apparent clustering over large distances.

If we assume a Gaussian-shaped form of the PSF with a standard deviation of ó, the normalized 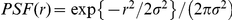 and 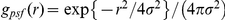. In this case, 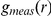 becomes:
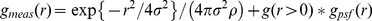
(1)The first term of 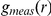 arises from over-counting of labeled molecules with finite resolution and is inversely proportional to the average density of labeled molecules (ρ). The second term describes the distribution of labeled molecules within the resolution limits imposed by the average PSF and is independent of the density of labeled molecules. This is graphically depicted in Figure 1 for the example of labeled molecules partitioned either randomly or into circular domains. In the special case of a random distribution of labeled molecules, 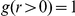 and
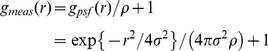
(2)For comparison, another methodology commonly used to quantify heterogeneity in labeled membrane systems is the modified Ripley's K function, denoted 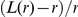. 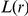 is related to the average number of signals within a radius r of a given particle [10], which is the integral of 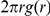. As a result, Ripley's methods are not well suited to quantify images that are subject to over-counting, since over-counting at short distances is propagated to long distances through the integration. By contrast, the correlation function is not much affected by over-counting when evaluated at distances larger than the width of the PSF, as demonstrated by comparison of Figures 1C and 1E. The mathematical relationship between 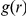 and 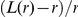 used to generate the curves in Figure 1E is presented in Materials and Methods.

### Some considerations when estimating the magnitude of apparent clustering

The estimates of apparent clustering due to over-counting that are presented in the first terms of Eqns. 1 and 2 are valid only when over-counting occurs via a random process. More rigorously, this applies when the number of times a given labeled molecule is sampled is well approximated by a Poisson distribution. This is expected to be the case for the majority of high-resolution measurements that are subject to over-counting, such as stochastic blinking of fluorophores in STORM/dSTORM measurements and reversible switching of fluorescent proteins in some PALM/fPALM measurements. This case should also apply when over-counting occurs through conjugation of multiple organic fluorophores to proteins or ligands, or when labeling of proteins with primary and secondary antibodies. As has been documented previously by others, these equations also hold in diffraction limited images in the limit where an ensemble of photons samples the PSF of each observed fluorophore and similar properties of measured correlation functions have been exploited to extract the oligomizeration state of labeled molecules [11].

Our estimates of clustering will not be accurate if over-counting is not randomly distributed over all labeled molecules. The first terms of Eqns. 1 and 2 will over-estimate apparent clustering from over-counting for cases where labeled molecules are sampled less frequently than expected from a Poisson distribution. This would occur, for example, when detection of a signal from a labeled molecule decreases the probability that the same labeled molecule will be detected additional times. This occurs in super-resolution fluorescence localization measurements if there is a significant probability of bleaching a fluorophore after it is activated. If, in fact, imaging is conducted in a manner that ensures that all labeled molecules are counted at most once, then measured correlations are due only to clustering of labeled molecules, and over-counting is not a problem. This is the ideal case for PALM/fPALM measurements if every activated fluorophore is irreversibly bleached after being counted, or for EM measurements if a labeling strategy is employed that ensures at most a single gold particle label per target protein. We note that several recent studies have demonstrated that some popular ‘irreversible’ PALM/fPALM probes show reversible blinking under some imaging conditions [9], [12], [13]. Our estimates of clustering will also not be accurate if there is significant noise in the image. Noise in the form of incorrectly identified signals or nonspecific labeling would act to decrease the magnitude of all correlations.

The first terms of Eqns. 1 and 2 will underestimate the magnitude of apparent clustering when labeled molecules are sampled more frequently than expected from a Poisson distribution. This would occur, for example, when the act of counting a signal from a labeled molecule increases the probability that additional signals will be detected from the same labeled molecule. This condition occurs in super-resolution fluorescence localization measurements if activated probes are counted once for each frame in which they are imaged, including cases when the same signal remains activated in multiple sequential image frames. A rigorous derivation demonstrating how deviations from a Poisson distribution quantitatively alter the magnitude of the over-counting term can be found in Materials and Methods.

### Deliberate over-counting quantifies effective resolution

**Figure 2:**
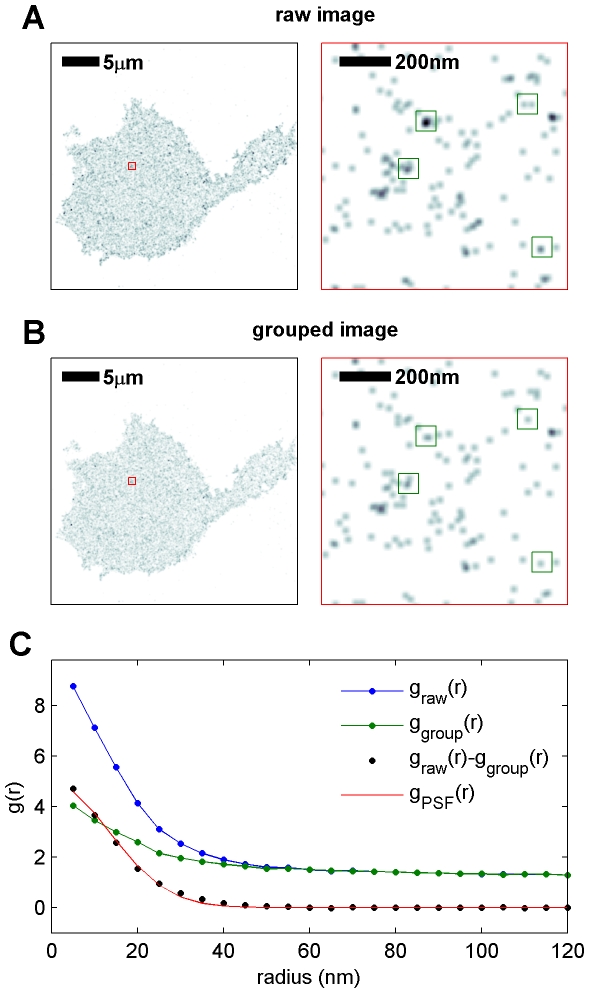
Measuring effective resolution of reconstructed super-resolution images with explicit over-counting. (A,B) Reconstructed super-resolution fluorescence localization images of labeled IgE on the bottom surface of RBL-2H3 mast cells. The region enclosed in the red box is magnified in the right panel. The image shown in A is reconstructed from raw data where each localized signal is counted independently. In B, intentional over-counting arising from probes remaining activated for multiple sequential frames is removed by grouping localized signals found at the same location within a small radius in sequential raw images. Grouping methods are described in Materials and Methods, and several locations which differ between the grouped and raw images are highlighted with green squares in the zoomed images. (C) Correlation functions are calculated from both the raw image to obtain 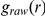 and from the grouped image to obtain 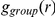. The correlation function of the raw image contains more apparent clustering at short radii than the measured correlation function of the grouped image because there are additional contributions in the raw image from intentional over-counting. Subtracting 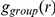 from 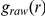 results in a curve that is proportional to the correlation function of the effective point spread function, 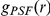. This is a measure of the effective resolution of the measurement. In this example, the black points are fit assuming a Gaussian PSF, 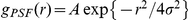, where σ is determined to be 9.6 nm and A = 4.9 is an constant related to the average number of times each probe was deliberately over-counted. In A and B, images on the left are filtered with a Gaussian PSF with standard deviation of 75 nm and zoomed images on the right are filtered with a Gaussian PSF with standard deviation of 10 nm for display purposes.

Deliberately over-counting probes is useful for isolating the over-counting term in Eqn. 1 and thereby directly measuring the effective average PSF of the measurement. An example of this approach is shown in Figure 2 for the case of a reconstructed super-resolution fluorescence localization image of labeled IgE-FcεRI on the RBL cell surface. We isolate the autocorrelation of the average PSF of the measurement, 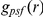, by first tabulating correlation functions from two images reconstructed from the same set of localized single molecule centers (signals). The first image is shown in Figure 2A and is reconstructed from intentionally over-counted signals (i.e. where signals localized in the same position in sequential frames are counted independently), whereas the second image shown in Figure 2B is reconstructed from signals where over-counting is avoided by grouping signals that occur within some small distance in sequential observations. Subtracting 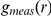 of the grouped image from 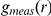 of the intentionally over-counted image results in a curve that is proportional to 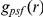, as the second term of Eqn. 1 is independent of the number of times a labeled molecule is counted. This is shown in Figure 2C. Note that in this example, both the raw and grouped measured correlation functions do not go to 1 at the largest radii shown in Figure 2C (r = 120 nm). This is because, for demonstration purposes, the entire image was used to calculate the measured correlation function and the majority of the image intensity is localized within the cell that extends for many microns, leading to long range contributions to 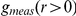. These contributions are not present in 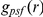. All remaining correlation functions presented in subsequent figures are tabulated using only contiguous regions of the cell membrane, as described in Materials and Methods.

In an ideal experiment, the range of 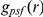 will be simply related to the average localization precision of acquired signals. In many cases, this calculated 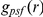 will be broader than the average localization precision extracted from fitting single fluorophores because it also contains contributions from limitations that are not explicitly accounted for in the experiment. Such factors could include incomplete correction for stage drift, finite mobility of labeled molecules [14], or inadvertent grouping of distinct fluorophores. This method will not produce accurate effective resolutions if sequential occurrences of the same fluorophore are not appropriately grouped (e.g. if the grouping radius is too small), if immobilized probes are incorrectly localized due to orientation effects on fluorescence emission [15], or if artifacts that reduce resolution occur on time-scales much longer than the lifetime of activated fluorophores.

### Pair correlation functions quantify heterogeneity

**Figure 3:**
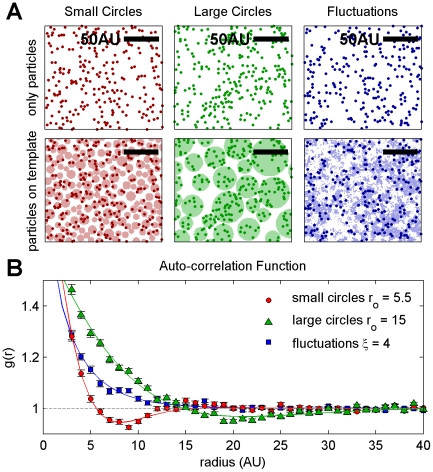
Correlation functions quantify heterogeneity. A) Simulated particle distributions are created by placing particles with radii of two arbitrary units (AU) at random on pre-made templates. Three examples are shown: small circles have radii between 4 AU and 8 AU (left), large circles have radii between 10 AU and 30 AU (center), and fluctuations are produced by simulating an Ising model at T = 1.075 T_c_ (right), where T_c_ is the critical temperature and the predicted correlation length (ξ) is ∼4 AU [19]. The top and bottom panels under each heading in A display the same particle distributions, while the bottom panels in A show both the particles and the template for demonstration purposes. Correlation functions are tabulated from a large number of simulations resembling the ones shown in the top panels (A). The correlation functions in B are fit to two different functional forms to account for distinct features in the curves. g(r) for the two circle distributions have a well defined dip below g(r) = 1, and are fit to a damped cosine function: g(r) = 1+A×exp(−r/α)×cos(πr/2r_o_), where A is an amplitude, α is a measure of the coherence length between circles, and r_o_ is the average circle radius. This is the predicted functional form for a correlation function of a micro-emulsion [18]. The correlation function to the fluctuation model does not dip below g(r) = 1 and is fit to the predicted form for critical systems: g(r) = 1+A×r^−1/4^×exp(−r/ξ). From this example, it is apparent that both the shape and range of the correlation function can reveal significant information regarding the underlying structure that gives rise to the heterogeneity. Also, when correlation functions are fit to the appropriate model, they accurately reproduce the radii of the circle distributions and the correlation length of the fluctuating distribution shown in part A.

For cases in which measured correlation functions contain contributions that cannot be attributed to over-counting, such as when 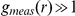 for 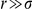, then the residual correlations can be attributed to clustering of labeled molecules. Much information can be extracted to discern the underlying structural distribution by monitoring both the shape and the magnitude of the correlation function. For example, the number of labeled molecules that are clustered together on average is given by 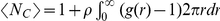, and the effective potential of mean force (PMF) between labeled molecules is given by 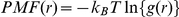 [16]. The shape of the correlation function also sheds light on the physical basis that governs heterogeneity [17]. Three examples of different simulated particle distributions are shown in Figure 3A, and their calculated correlation functions shown in Figure 3B have distinct features that can be used to distinguish the organizing principles giving rise to these distributions. Simulations of particles placed within a series of circular domains produce correlation functions that are damped oscillations, where the frequency of the oscillations corresponds to the average domain size, and the decay length quantifies correlations between neighboring domains [18]. By contrast, simulations of particles distributed in fluctuations produce correlation functions that decay as exponentials [19]. Both micro-emulsion (circles) and fluctuation models have been proposed as physical mechanisms that could produce small and subtle heterogeneity in resting cell plasma membranes [20], [21], and, in principle, the shapes of correlation functions can be used to distinguish these different models.

### Over-counting in super-resolution fluorescence localization images

**Figure 4:**
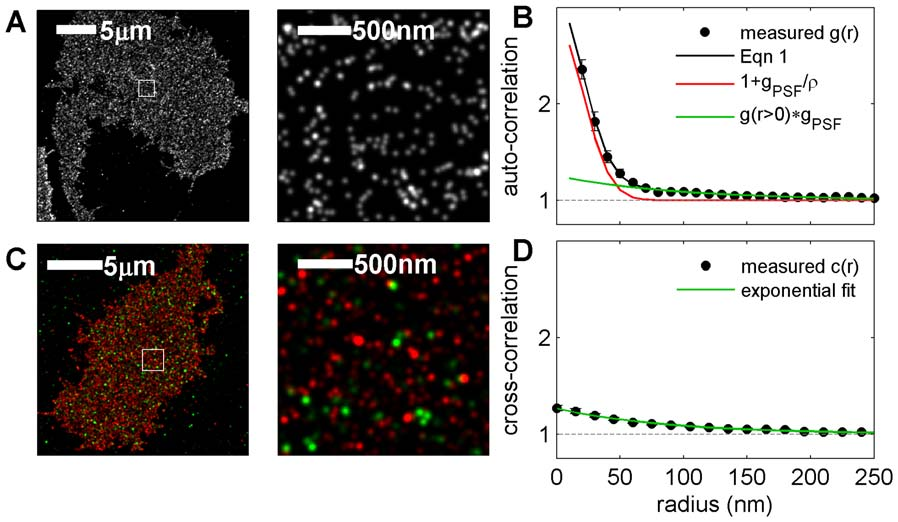
Apparent clustering of IgE-FcεRI observed using super-resolution fluorescence localization imaging is dominated by over counting of individual labeled protein complexes. (A) Reconstructed super-resolution fluorescence localization image of a representative RBL-2H3 cell fixed after labeling with IgE directly conjugated to Alexa-647. Magnification of square inset shown at right. Localized centers are convolved with a Gaussian PSF with σ = 50 nm (whole cell) or σ = 20 nm (inset) for display purposes. (B) Correlation functions of localized single molecule centers averaged over 8 cells are fit well by Eqn 1 for 30 nm<*r*<500 nm assuming an exponential form of 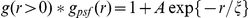. Error bars on black points represent the standard deviation of the mean of the 8 cells. Extracted fit parameters are: σ = 21±1 nm, ρ = 200±6 µm^−2^, A = 0.25±.03, and ξ = 95±8 nm. (C) Reconstructed super-resolution fluorescence localization image of a representative RBL-2H3 cell fixed after labeling with two distinct pools of IgE, one directly conjugated to Alexa-647 (red) and the other directly conjugated to Alexa-532 (green). As in A, localized centers are convolved with a Gaussian PSF with σ = 50 nm (whole cell) or σ = 20 nm (inset). (D) Cross-correlation functions of localized single molecule centers between the two colors are averaged over 6 cells, and error bars represent the standard error of the mean between cells. The measured cross-correlation function is well fit for *r*<450 nm by a single exponential, 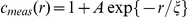. Extracted fit parameters are A = 0.26±.02, and ξ = 89±6 nm, in good agreement with the parameters obtained by fitting the auto-correlation function in the single color experiment.

We apply this correlation analysis to two types of super-resolution data obtained with labeled IgE specifically bound to the high affinity FcεRI receptor on RBL-2H3 mast cells. Figure 4A shows a reconstructed super-resolution fluorescence localization image of Alexa-647 fluorophores conjugated directly to IgE on the ventral (bottom) surface of a chemically fixed cell. In these measurements, the majority of probes are forced into a reversible dark state in the presence of bright light, a reducing environment, and basic pH [4], [5]. This enables imaging and localization of a sparse subset of fluorophores at any given time. Probes stochastically switch between bright and dark states, and high resolution images are reconstructed from samples imaged over time, as described in Materials and Methods.

Correlation functions derived from images of localized single molecules from cells labeled with Alexa-647 conjugated IgE show significant auto-correlations at short distances and weak correlations that extend to longer distances, as shown in Figure 4B. We fit this measured correlation function to Eqn. 1 by approximating 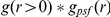 as a single exponential given by 
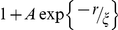
, where A is the amplitude and î describes the size of the structure. The best fit value for the average surface density (ρ) of labeled IgE is ρ = 200±6 µm^−2^, which is in good agreement with previous studies [22]. The short range auto-correlation (red curve) arises from over-counting as confirmed by cross-correlation analysis (see below). The long range auto-correlation (green curve) can be fit to obtain an amplitude of A = 0.25±.03 and a range of ξ = 95±8 nm.

Strong evidence that the large correlations at short radii arise from over-counting labels on single IgE-FcεRI complexes and not from self-clustering of proteins is provided by measurements of cross-correlation functions calculated from two-color images (Figure 4C,D). Similar to auto-correlation, the cross-correlation function, c(r), quantifies the increased probability of finding a signal a distance r away from a given signal of a different type. Unlike the auto-correlation function, the cross-correlation function does not contain a delta function at *r = 0*, and therefore it is not affected by over-counting, even when an experiment is conducted with finite resolution. A detailed derivation of this statement is included in Materials and Methods. In the two-color experiment, we created two separate pools of FcεRI on the cell surface by pre-incubating cells with a mixture of IgE labeled with either the fluorophore Alexa647 or the fluorophore Alexa532 prior to fixation. Importantly, by this scheme, both species of fluorophore cannot label the same FcεRI protein because only a single IgE antibody binds to each FcεRI protein [23]. After cell fixation, each color channel was imaged sequentially. Final reconstructed images of the different color channels are merged with the aid of fiduciary markers for accurate alignment (Figure 4C).

Measured cross-correlation functions lack the large correlations at short distances that dominate auto-correlations functions tabulated from single color images (Figure 4B), but they retain the weak correlations at larger radii (Figure 4D). This measurement confirms that large clustering at short radii arises from over-counting IgE-FcεRI complexes in auto-correlated, single-label experiments. Fitting measured cross-correlation functions to an exponential function 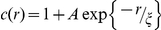 yields an amplitude of A = 0.26±.02 and a range of ξ = 89±6 nm. Both parameters are in good agreement with those extracted from fitting the auto-correlation function in Figure 4B after isolating contributions from over-counting as described above.

The magnitude of measured cross-correlation functions suggests that IgE-FcεRI clustering arises from a thermally driven mechanism, since 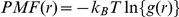 indicates that the potential of mean force is on the order of 1k_B_T. The shape of the measured cross-correlation function is well fit to an exponential and does not appear to drop below 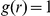. This is consistent with an irregular structure that more closely resembles the image of fluctuations than the images of circles in Figure 3. These measured auto-correlation and cross-correlation functions are consistent with our recent theoretical predictions of critical fluctuations in plasma membranes at physiological temperatures [20], [24], although it is equally possible that weak correlations arise from other mechanisms such as undulating membrane topology or interactions with the glass substrate.

### Over-counting in scanning electron microscopy images

**Figure 5:**
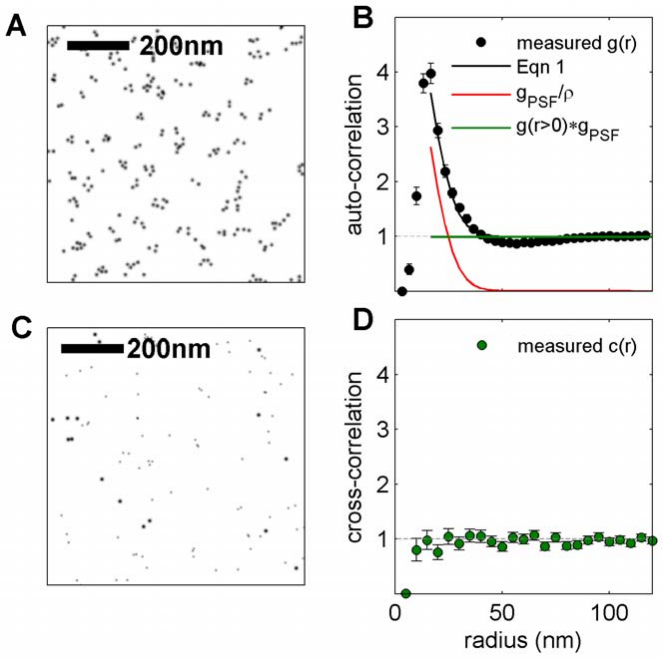
Apparent clustering of IgE-FcεRI observed using immuno-gold labeled SEM is dominated by multiple gold particles binding to single target proteins. (A) A reconstructed image showing gold particles labeling IgE-FcεRI complexes on the top surface of a representative fixed RBL-2H3 cell. IgE-FcεRI is labeled post fixation with primary and gold-tagged secondary antibodies. (B) Auto-correlation functions, g(r) are averaged over 80 distinct SEM images, and error bounds describe the standard error of the mean. Fits of g(r) of to Eqn 1 for radii between 20 nm and 150 nm are consistent with g(r>0) = 1, indicating that any self-clustering of IgE-FcεRI cannot be distinguished from clustering arising from over-counting. Extracted fit parameters are σ = 13±0.5 nm for the standard deviation of the effective PSF and ρ = 157±5 µm^−2^ for the surface density of labeled IgE-FcεRI complexes. The average surface density of gold particles is 280 golds/µm^2^. (C) 10 nm and 5 nm gold particles label distinct populations of IgE-FcεRI in double label experiments. (D) Cross-correlation functions, c(r), are calculated using localized centers of the differently sized particles and are averaged over 18 distinct SEM images. Errors bars represent the standard error of the mean for c(r) curves tabulated from different images. Cross-correlation functions are not affected by over-counting and show no evidence for IgE self-clustering within error bounds. In parts B and D, depletion of correlation functions for r<10 nm arises from packing constraints of gold particles.

This correlation analysis can also be applied to scanning electron microscopy (SEM) images where target proteins are labeled with primary antibodies followed by secondary antibodies conjugated to gold particles as described in Materials and Methods. Figure 5 shows a flat section of the top surface of a RBL-2H3 cell with IgE-FcεRI complexes that are immuno-labeled with 10 nm gold particles. This labeling scheme allows for multiple gold particles to decorate individual target proteins, and the correlation function detects clustering over short distances (Figure 5B). In this experiment, the PSF is governed by the finite size of labeling antibodies and gold particles and not by the precision of localizing the gold particle centers. Measured correlation functions tabulated from images of gold particle centers show depletion at very short radii, 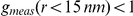, because the gold particles cannot pack closer than their hard sphere radius. Fitting the measured auto-correlation function to either Eqn. 1 or 2 yields σ = 13±0.5 nm and ρ = 157±5 µm^−2^. This surface density is comparable but somewhat lower than that calculated from our fluorescence measurements, but still within expected values [22]. It is possible that this extracted surface density of IgE-FcεRI underestimates the actual surface density of complexes, since labeling of gold particles may not be well approximated by a Poisson distribution due to the large size of gold particle labels.

Direct evidence that apparent clustering of labeled IgE-FcεRI complexes is dominated by contributions from over-counting is provided by double-label SEM experiments, where distinguishable but functionally identical pools of IgE-FcεRI are labeled with differently sized gold particles (Figure 5C). Just as in our double label fluorescence experiments, this measurement was conducted by first creating two separate pools of FcεRI on the cell surface by pre-incubating the cells with a mixture of IgE labeled with either the fluorophore Alexa488 or the fluorophore FITC prior to fixation. These were distinctively labeled with fluorophore-specific primary antibodies of different species followed by species-specific secondary antibodies conjugated to gold particles of different sizes (Figure 5C). By this scheme, small and large gold particles cannot bind to the same FcεRI protein. We find that cross-correlation functions tabulated between differently sized particles indicate random distributions within experimental error bounds (Figure 5D). This comparison shows that the appearance of clustering in single label images (Figure 5B) is dominated by over-counting individual target proteins.

Thus, unlike our super-resolution fluorescence localization measurements (Figure 4), we do not detect significant self-clustering over longer distances when we visualize gold labeled proteins using SEM. This could be because we selected morphologically flat regions of the cell surface for our SEM measurements (see Materials and Methods), while we could not independently measure surface topology in our fluorescence measurements. Another possible reason for the difference could be that receptors are organized differently on the top and bottom surfaces of the cell. SEM measurements were acquired from the top (dorsal) cell surface, while the fluorescence images were acquired from the bottom (ventral) cell surface.

Our analysis of both super-resolution fluorescence localization and SEM images yields results that differ from those of several previous studies which report that IgE-FcεRI complexes are tightly pre-clustered into small domains in unstimulated RBL-2H3 cells by electron microscopy [25], [26], [27]. Since similar strategies were used to label IgE-FcεRI in these studies, we expect that over-counting of IgE-FcεRI complexes was incorrectly identified as self-clustering of these target proteins. It is possible that previous reports of self-clustering of other membrane components visualized by electron microscopy can also be attributed to over-counting, since labeling schemes often require the use of multiple or polyclonal antibodies. This potential pitfall of electron microscopy labeling and imaging was noted in early work that contributed to the Fluid Mosaic Model of biological membranes [28].

### Quantifying receptor clustering and over-counting in SEM images

**Figure 6:**
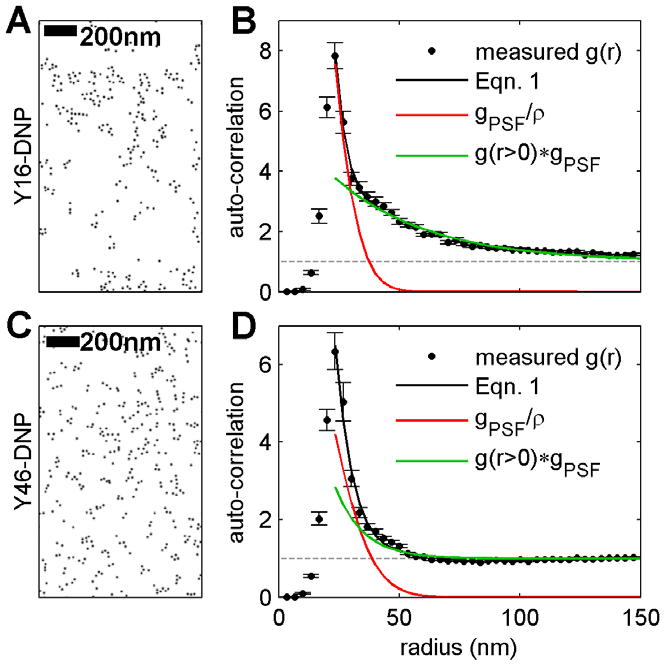
Clustering of YDNA ligand-bound IgE-FcεRI complexes imaged using SEM shows clustering both from over-counting and extended protein domains. (A,C) Reconstructed gold particle centers labeling IgE-FcεRI from a representative SEM image of an RBL cell surface that has been stimulated for 10 min with the trivalent YDNA ligands Y16-DNP (A) and Y46-DNP(C). (B, D) Measured correlation functions from YDNA treated cells include contributions from over-counting and extended clustering, and are well fit by Eqn 1 for radii between 25 nm and 160 nm assuming an exponential form of 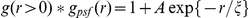. In B, the correlation function is an average 23 individual SEM images, and in D the average is over 40 SEM images, and in both cases error bars represent the standard error of the mean between images. In Y16-DNP treated cells, we observe extended domains and the extracted fit parameters are: σ = 10±1 nm, ρ = 27±4 µm^−2^, A = 5±0.4, and ξ = 39±2 nm. The average surface density of gold particles labeling IgE is 107 golds/µm^2^. Gold particles labeling IgE-FcεRI in Y46-DNP treated cells appear to be clustered into smaller structures, as reflected in the fit of the measured correlation function to Eqn 1, with extracted fit parameters: σ = 13±1 nm, ρ = 50±23 µm^−2^, A = 13±29, and ξ = 11±5 nm, and the average surface density of gold particles labeling IgE is 148 golds/µm^2^. Note that the errors associated with fit parameters are significantly larger in the case of Y46-DNP treated cells compared to Y16-DNP treated cells because the observed structure is of a size that is comparable to the effective PSF of the SEM measurement.

Large-scale clustering of IgE-FcεRI is observed when cells are treated with a multivalent antigen that crosslinks multiple surface-bound IgE antibodies. Figure 6 shows reconstructed SEM micrographs of RBL cells treated for 10 minutes with trivalent dinitrophenyl (DNP) ligands. These architecturally defined ligands are based on a Y-shaped, DNA scaffold with DNP groups conjugated to each of the three 5′ ends. The distance between DNP molecules is set by the number of bases in each of the complementary single strands that are annealed to form the double stranded Y-structure, and for Y16-DNP and Y46-DNP that distance is 5±1 nm and 13±2 nm, respectively [29]. Because the anti-DNP IgE used in these experiments contain two DNP binding sites, the trivalent Y-DNP ligands can cross-link IgE-FcεRI complexes into branched clusters.

Gold particles labeling IgE-FcεRI from cells incubated for 10 min with Y16-DNP show clear extended clusters in reconstructed SEM images (Figure 6A), and this structure is reflected in measured auto-correlation functions (Figure 6B). Correlation functions from Y16-DNP treated cells are well fit by Eqn 1, assuming an exponential form of 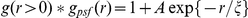, and extracted fit parameters are given in the caption to Figure 6. The average dimensions of the clusters (ξ = 39±2 nm) is much larger than the width of the effective PSF (σ = 10±1 nm), and this provides confidence in the fit of both the long-range and short-range components of the data. However, the best fit value for surface density is ρ = 27±4 µm^−2^, which is significantly lower than our anticipated surface density of IgE-FcεRI complexes and well below our measured gold surface density of 107 golds/µm^2^. It is likely that the peak at short radius also contains contributions from IgE-FcεRI complexes organized into small oligomers as a result of exposure to crosslinking ligand. In this case, we can interpret the best fit surface density to represent the surface density of small oligomers. If we assume that the actual surface density of IgE-FcεRI is well approximated by the surface density of gold labels, then we would conclude that IgE is organized into tetramers on average. It is also possible that the gold surface density over-estimates (or under-estimates) the IgE-FcεRI surface density and complexes are organized into trimers (or pentamers) on average. Unfortunately, we do not explicitly know the surface density of IgE under this stimulation condition and it is not possible to clearly distinguish small protein clusters from over-counting in single label experiments.

Extended clusters are less apparent in reconstructed images of gold labeled IgE-FcεRI complexes in cells incubated for 10 min with the larger Y46-DNP ligand (Figure 6C). Auto-correlation functions tabulated from these images are shown in Figure 6D and can also be fit to Eqn. 2 assuming an exponential form of 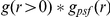. In this example, extracted fit parameters cannot be determined with confidence because the size of extended structures (ξ = 11±5 nm) are comparable to the extracted width of the PSF (σ = 13±1 nm). We also find that the extracted surface density (ρ = 50±23 µm^−2^) is much lower than the measured surface density of gold particles labeling IgE (148 µm^−2^), again suggesting the presence of small IgE-FcεRI oligomers on the cell surface. If the surface density of IgE-FcεRI complexes is well approximated by the surface density of gold particles, then we would conclude that receptor complexes are organized primarily as trimers. Unfortunately we cannot draw quantitative conclusions since we do not have independent measurements of receptor surface density under these conditions. Our previous studies showed that Y46-DNP stimulates less cell activation than Y16-DNP, consistent with the lower amount of extended clustering of IgE-FcεRI with the former that is revealed in these images [29].

In conclusion, we demonstrate that correlation functions provide an analytical tool to quantify heterogeneous distributions of labeled molecules in super-resolution experiments, even in the presence of over-counting that gives rise to the artifactual appearance of short-range clustering. We present an analytical method that predicts the magnitude of correlations arising from over-counting, and we describe a procedure to measure the apparent PSF of an image for cases when signals can be intentionally over-counted. We have validated this analysis methodology by quantifying the lateral distribution of IgE-FcεRI complexes on the surface of unstimulated RBL-2H3 cells imaged using super-resolution fluorescence localization and SEM. We detect weak clustering of IgE-FcεRI complexes when imaged on the ventral cell surface using TIRFM and super-resolution fluorescence localization methods, and these complexes appear randomly distributed when imaged on flat areas of the dorsal surface by SEM. Our interpretations of single-labeled IgE-FcεRI images are confirmed by direct measurements of cross-correlation functions in double label experiments using both imaging methods. We additionally quantify over-counting and long-range clustering in cells that have been stimulated using defined Y-DNP ligands and discuss the advantages and limitations of applying this correlation method to interpret clustered distributions of proteins. These examples emphasize the importance of explicitly considering over-counting when quantifying images of proteins in membranes, where the extent of heterogeneity may be small and subtle.

## Materials and Methods

### Chemicals and Reagents

FITC, Alexafluors 647, 532, 488, and rabbit anti-Alexafluor 488 were purchased from Invitrogen (Eugene, OR). Mouse anti-FITC, 10 nm gold-conjugated anti-rabbit IgG (whole molecule), 10 nm gold-conjugated anti-mouse IgG (whole molecule), 5 nm gold-conjugated anti-rabbit IgG (whole molecule), β-mercaptoethanol, Glucose Oxidase, and Catalase were purchased from Sigma (St. Louis, MO). 5 nm gold-conjugated anti-mouse was purchased from GE Healthcare (Piscataway, NJ). A488-IgE, A532-IgE, A647-IgE, and FITC-IgE were prepared by conjugating purified mouse monoclonal anti-2,4-dinitrophenyl (DNP) IgE with Alexafluor 488, Alexafluor 532, Alexafluor 647, or FITC as previously described [30], [31]. Trivalent Y-shaped, double stranded DNA ligands, Y16-DNP and Y46-DNP, were prepared as described previously [29]. Glutaraldehyde (25% stock) was purchased from Ted Pella (Redding, CA). Para-formaldeyde was purchased from Electron Microscopy Services (Hatfield, PA).

### Super-resolution fluorescence localization imaging

#### Sample preparation

Rat Basophilic Leukemia (RBL-2H3) cells were cultured as described previously [30], then harvested using Trypsin-EDTA, and plated sparsely overnight at 37°C in glass-bottom MatTek dishes (Ashland, MA). The cells were sensitized with either A647-labeled IgE (1 µg/ml) (for single color experiments) or a mixture of A647-labeled IgE and A532-labeled IgE (1 µg/ml total) (for two color experiments) in HEPES buffered media for 1 to 2 hours at room temperature. Dishes containing cells were rinsed, incubated in media at 37°C for 5 minutes, rinsed again with warm PBS, and were then chemically fixed (4% paraformaldehyde 0.1% glutaraldehyde in PBS) for 10 minutes at room temperature. Samples were then blocked with 2% fish gelatin, 2 mg/mL BSA in PBS for 10 minutes.

#### Imaging

**Figure 7:**
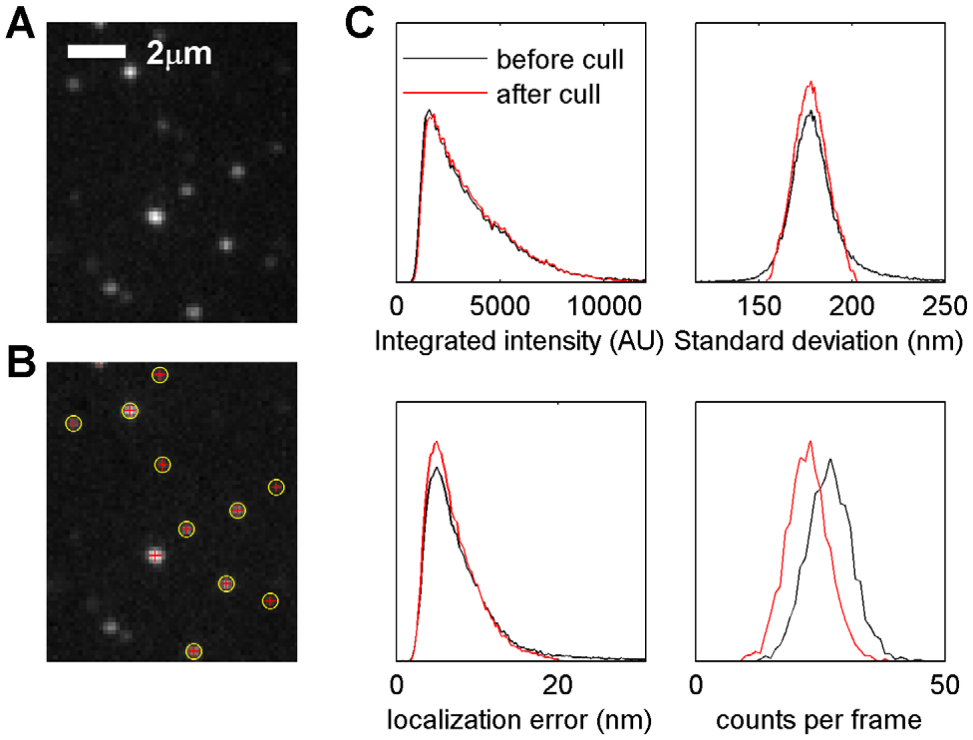
Culling of super-resolution fluorescence localization data is accomplished using distributions of parameters extracted from fitting single diffraction limited spots. (A) An example unprocessed fluorescence image showing an array of diffraction limited spots of Alexa647 probes bound to IgE. This is a raw data image for the cell shown in Figure 2. (B) A background subtracted image for the same data shown in part A showing localized centers. Background is evaluated by averaging over 500 sequentially acquired images. Diffraction limited spots that are fit to 2D Gaussian functions are shown as red crosses, where the length of the cross is given by the best fit standard deviation. Localized spots that are included for analysis after the culling procedure are also labeled with yellow circles. (C) Normalized histograms showing the distribution of fit parameters obtained from a population of fits before (black lines) and after (red lines) the culling procedure. The integration time is longer than the lifetime of the active state of most fluorophores observed, and this likely contributes to the skewed distribution of integrated intensities in this experiment. The best fit standard deviation (σ) is normally distributed around 177 nm. This distribution is fit to a 1D Gaussian with standard deviation *s* and culled to only include values that are consistent with σ = <σ>±1.5 *s*. Localized fits with larger localization errors are also culled. These culling steps result in a smaller number of localized diffraction limited spots per frame. Over 2500 frames, 67053 single diffraction limited spots were fit, of which 56101 (83%) were included after culling.

Single label samples were imaged on an inverted microscope (Leica DM-IRB, Wetzlar, Germany) under through-objective TIRF illumination by a 100 mW 642 nm diode pumped solid state (DPSS) laser (Crystalaser, Reno, NV). Double label experiments were conducted on an inverted Olympus IX81-ZDC microscope with a cellTIRF module (Olympus America, Center Valley, PA) under through-objective TIRF illumination by either a 75 mW 642 nm DPSS laser (Coherent, Santa Clara, CA) or a 150 mW DPSS 532 laser (Cobolt, Stockholm, Sweden). In both cases, images were captured with an Andor iXon 897 EM-CCD camera (Belfast, UK) using custom image acquisition code written in Matlab (Mathworks, Natick, MA). To induce A647 or A532 photo-switching, cells were imaged in the presence of an oxygen-scavenging and reducing buffer containing 100 mM Tris, 10 mM NaCl, 10% w/w glucose, 500 µg/mL glucose-oxidase, 40 µg/mL catalase, and 1% β-mercaptoethanol at pH 8. Movies of A647 or A532 photo-switching were acquired at between 5 and 25 frames per second for at least 2500 frames and analyzed by localizing the centers of diffraction limited spots through least squares fitting a two dimensional Gaussian shape using the *fminfunc()* function in Matlab. An example image with fits is shown in Figure 7A–B. Localized centers were culled to exclude outliers in standard deviation and localization precision in an effort to remove contributions from multiple emitters and poorly fit diffraction limited spots. Culled events are not correlated in space, and statistics for a typical example are shown in Figure 7C. We find that the fit parameters width and localization precision of diffraction limited spots are normally distributed around expected values, while brightness follows a skewed distribution, as has been noted previously [32]. Localized centers were combined (grouped) in single label measurements when the same fluorophore was identified in sequential images at the same position within twice the maximum allowed localization precision of the population of fits. This grouping is done to minimize intentional over-counting of single fluorophores in single color experiments. No grouping was done in two color measurements. Reconstructed images are assembled by incrementing a pixel value once for each time that a localized signal is identified at that location. Correlation functions are tabulated from these unfiltered reconstructed images. For display purposes, reconstructed images are filtered with a Gaussian PSF as indicated in the figure captions.

### Scanning electron microscopy (SEM)

#### Sample Preparation

RBL-2H3 mast cells were grown overnight to ∼50% confluency on 2 mm×2 mm silicon chips at 37°C under standard cell culture conditions [33], and high affinity IgE receptors (FcεRI) were labeled with either A488-IgE (1 µg/mL) (for single label experiments) or a 1∶1 mixture of A488-IgE and FITC-IgE (total 1 µg/mL) (for double label experiments) for 2–3 hr prior to the experiment. Cells were washed quickly in phosphate buffered saline (PBS), and immediately fixed in 4% (w/v) p-formaldehyde and 0.1% (w/v) glutaraldehyde for 10 min at room temperature in PBS. Fixed cell samples were washed in blocking solution (2 mg/mL BSA and 2% (v/v) fish gelatin in PBS) and labeled sequentially with primary antibodies and gold conjugated secondary antibodies in blocking solution. Incubations were 1 h at room temperature with wash steps in between. After labeling, the cell samples were further fixed in 4% p-formaldehyde and 1% glutaraldehyde for 5 min at room temperature, and then thoroughly washed in distilled water. Following dehydration through a series of graded ethanol washing steps, samples were critical point dried, mounted on round aluminum SEM stubs, and sputtered with carbon to prevent charging. For single label experiments the primary antibody was rabbit anti-Alexafluor 488 and the 10 nm gold conjugated secondary antibody was goat anti-rabbit IgG. For double label experiments, the primary antibodies were mouse anti-FITC and rabbit anti-Alexafluor 488, while the secondary antibodies were 5 nm gold-conjugated anti-rabbit IgG and 10 nm gold-conjugated anti-mouse IgG. Samples were labeled first with 10 nm and then 5 nm gold antibody conjugates.

*Imaging*: Mounted samples were imaged with a Schottky field emission Scanning Electron Microscope (LEO 1550) at 20 KeV. The dorsal (top) surfaces of intact, adherent cells were imaged using secondary electron detection (SED) and backscattered detection (BSD) at high magnification. Flat membrane regions were selected for imaging. For imaging 10 nm gold particles, individual micrographs were obtained at 35 K magnification, and typical images cover 2.4 µm^2^ of the cell surface. For imaging 5 nm gold particles and in double-label experiments with 10 and 5 nm gold particles, micrographs were obtained at 75 K–100 K magnification. Immuno-gold labeled protein distributions for ≥10 different cells and ≥2 individual experiments were obtained for all experimental conditions presented. Gold particle centers were localized by finding the weighted centroid of identified particles using automated image processing software written in Matlab. Correlation functions were tabulated from these binary images of gold centers. Reconstructed images are formed by convolving an image of the particle centers with a Gaussian shape with half-width given by the gold particle radius.

### Calculation of correlation functions

Pair auto-correlation functions were tabulated in Matlab using Fast Fourier Transforms (FFTs) as follows:
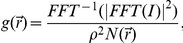
where 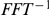 is an inverse Fast Fourier Transform and 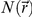 is a normalization that accounts for the finite size of the acquired image. In the case of super-resolution fluorescence localization measurements, *I* is the unfiltered reconstructed image of localized probes, generated as described above. For SEM measurements, *I* is a binary image of localized gold particle centers. In either case, the image *I* is padded with zeros in both directions out to a distance larger than the range of the desired correlation function (maximally the size of the original image) to avoid artifacts due to the periodic nature of FFT functions. The normalization factor 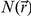 is the autocorrelation of a window function W that has the value of 1 inside the measurement area, and is also padded by an equal number of zeros.
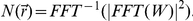
This normalization is essentially the total squared area over which the correlation function is calculated accounting for the fact that there fewer possible pairs separated by large distances due to the finite image size. When calculating correlation functions from reconstructed super-resolution fluorescence localization images, the cell interior was first masked, and this mask was then used as the window function W. The choice of the window function can impact the tabulated correlation function, and efforts were made to exclude regions of the cell periphery or regions with noticeable membrane topology. Under these conditions, the measured correlation functions do not depend strongly on the mask used.

Pair cross-correlation functions were computed using two images. In super-resolution fluorescence localization measurements, one image was reconstructed from localized Alexa 647 fluorophores (*I_1_*), while the second image was reconstructed from localized Alexa 532 fluorophores (*I_2_*). In SEM measurements, one image was reconstructed from the locations of 5 nm gold particle centers (*I_1_*) and the second image was reconstructed from locations of 10 nm gold particle centers (*I_2_*).
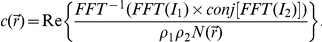
Here 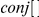 indicates a complex conjugate, ρ_1_ and ρ_2_ are the average surface densities of images *I_1_* and *I_2_* respectively, and Re{} indicates the real part. This computation method of tabulating pair auto and cross-correlations is mathematically identical to brute force averaging methods. Correlation functions were angularly averaged by first converting to polar coordinates using the Matlab command *cart2pol()*, and then binning by radius. 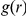 values are obtained by averaging 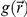 values that correspond to the assigned bins in radius. Errors in 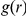 are dominated by counting statistics.

### Calculation of modified Ripley's K functions

The statistical significance of clustering can also be determined using the Ripley's K function, which measures the increased density of particles within a circle of radius r and is related to the pair correlation function through integration:
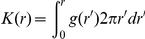
Frequently, Ripley's K function is restated when plotting the results from electron microscopy studies [34]:
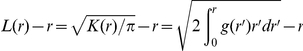
Furthermore, 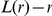 curves reported in the literature are typically normalized to a confidence interval, so that the amplitudes of normalized 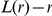 traces indicate the statistical significance of clustering within a radius r. Confidence intervals of 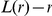 are calculated by propagating the statistical errors of 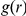 through 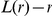 to obtain the curves presented in Figure 1E.

### Derivation of equations to estimate over-counting in pair auto-correlation functions

Below, we provide a detailed mathematical derivation of the equations used to analyze pair auto-correlation functions throughout the Results and Discussion section. First, we describe how to calculate a pair auto-correlation function of a collection of point particles. We then expand this to describe how this correlation function is modified when point particles are replaced by molecules that are sampled stochastically with finite resolution. We then take an expectation value of this stochastic auto-correlation function to obtain the equations used in the main text.

Consider a set of *N* point-like molecules at positions 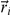 for 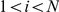 with average surface density 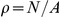, where 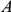 is the total area. The density of molecules as a function of 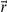 is given by 
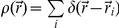
, where 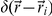 is a delta function at position 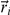.The exact correlation function of these molecules is given by:
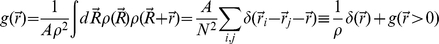
Where in the last step we have defined 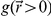 as the correlation function with only those terms where 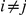. Note that this correlation function is normalized to 1 at spatial infinity, as defined in previous sections.

Now consider stochastically building this correlation function by taking repeated measurements of individual molecule positions with finite resolution. Such a measurement is stochastic in two respects. First, measurements stochastically sample the normalized effective point spread function 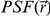. More rigorously, a particle located at position 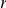 will be measured at 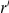 with a probability given by 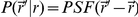. Second, the number of times that any given molecule is counted is itself stochastic. In this initial derivation we assume that individual measurements are uncorrelated, so that the number of times each molecule is sampled is governed by a Poisson distribution. When this assumption is valid, each measurement is taken independently from the distribution:
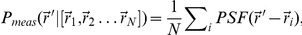
where N molecules are located at positions 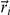 as described above. After making M of these measurements, the average measurement density is given by 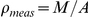 and we can construct a measured correlation function:
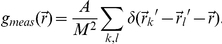
In this equation, *k* and *l* sum over measurements, and not molecules. This 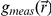 is stochastic even for a fixed positioning of underlying molecules, but we can relate its expectation value 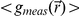 to the bare correlation function, 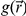 by averaging over the possible measurements of particle positions. Using the above assumptions for the probability distribution of each measurement, we calculate the expected value of 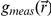 as follows:
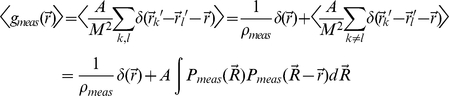
In the first line we have separated out terms where *k = l* and removed them from the expectation value. In the next line we note that each term appearing in the expectation value where 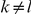 is proportional to the correlation function of the probability distribution of a single measurement with itself. Properly this term should be multiplied by a pre-factor of 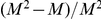 since we have removed terms where 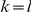, but we replace this with 1 in the limit where 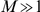. If we re-write the probability distribution in terms of the actual molecule positions *r_i_* in accordance with our form for 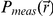, this expression becomes:
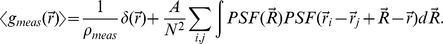
Using the definition of a convolution in two dimensions (denoted with a *) and defining 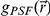 to be the correlation function of the point spread function with itself: 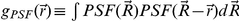, the expectation value for the measured correlation function can be written as:
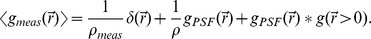
The only term in the above expression with a dependence on the density of measurements, 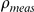, is the delta function centered at 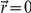 and arises from terms where *k = l*. This contribution is easily disregarded since it does not contribute to any values of 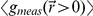. In contrast, we cannot easily distinguish the contribution that arises from duplicate measurements of the same molecule from measurements from distinct molecules. This happens for two reasons. First, we have no way of knowing whether two independent measurements (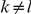) came from the same molecule (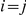). Second, the delta function that arises from including 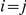 terms in 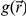 is spread over a PSF in 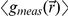 so that it becomes 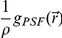. This term extends to finite radius and can no longer be easily distinguished from terms coming from the convolution of the point-spread function with 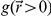.

### Modifications for cases where sampling of labeled molecules is not well approximated by a Poisson distribution

In the following section, we briefly discuss how these derivations would have to be modified if our assumption that each measurement is independent fails. In general, given a distribution, 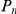, for the number of times, 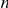, that each individual molecule is measured over the course of an experiment we expect to observe:
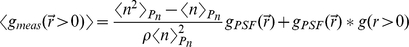
Where 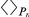 denotes the expectation value under the probability distribution 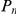. In a Poisson distribution 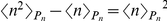 so that this equation reduces to the case derived in the text where we assumed that each measurement is independent. For cases where a subset of labeled molecules are sampled more frequently than expected from a Poisson distribution, then 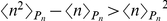, and the amplitude of the 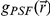 term of the measured correlation function will be greater than expected based in the measured surface density of labeled molecules. In contrast, when labeled molecules are sampled less frequently than expected from a Poisson distribution, then 
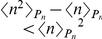
, and the amplitude of the 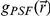 term of the measured correlation function will be smaller than expected based in the measured surface density of labeled molecules. If each particle is measured exactly zero or one time then 
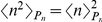
, and the measured correlation function becomes:
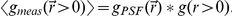
In this case, there is no longer any apparent clustering in 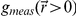 due to the over-counting.

### Modifications for measured cross-correlation functions

In this section, we briefly demonstrate important differences between measured pair auto-correlation functions and pair cross-correlation functions. An analogous calculation to the pair auto-correlation function described previously can be carried out for the pair cross-correlation function of two signals 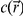. Given two distinguishable molecular types each located with centers at positions 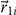 and 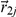 with 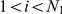 and 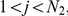 the cross correlation is defined by:
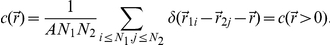
Note that the last equality stresses that there is no delta function contribution at the origin (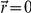). This is because *i* and *j* sum over different sets of distinguishable molecules and therefore terms where *i = j* do not represent cases where the same molecule is being detected by different signals. We note that this is only the case when a labeling scheme is employed that eliminates the possibility that two distinguishable probes label the same molecule. Carrying through an analogous calculation to the one previously described for 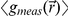 yields:
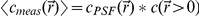
We use 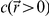 rather than 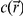 to stress that there is no artifacts due to over-counting and where the cross-correlation function of the distinguishable effective point spread functions is given by:
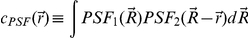
We note that 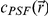 may differ from 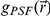 for each individual effective point spread function.

## Supporting Information

File S1 **A Matlab function to tabulate correlation functions from a two dimensional image.** To use, rename file as get_autocorr.m and call within a Matlab function, script, or at the command line. This function has been used successfully in Matlab version 2010a. Further information on function usage can be found within the file.
(M) Click here for additional data file.

## References

[1] Betzig E, Patterson GH, Sougrat R, Lindwasser OW, Olenych S (2006). Imaging intracellular fluorescent proteins at nanometer resolution.. Science.

[2] Hess ST, Girirajan TP, Mason MD (2006). Ultra-high resolution imaging by fluorescence photoactivation localization microscopy.. Biophys J.

[3] Klar TA, Jakobs S, Dyba M, Egner A, Hell SW (2000). Fluorescence microscopy with diffraction resolution barrier broken by stimulated emission.. Proc Natl Acad Sci U S A.

[4] Rust MJ, Bates M, Zhuang X (2006). Sub-diffraction-limit imaging by stochastic optical reconstruction microscopy (STORM).. Nat Methods.

[5] Heilemann M, van de Linde S, Schuttpelz M, Kasper R, Seefeldt B (2008). Subdiffraction-resolution fluorescence imaging with conventional fluorescent probes.. Angew Chem Int Ed Engl.

[6] Ji N, Shroff H, Zhong H, Betzig E (2008). Advances in the speed and resolution of light microscopy.. Curr Opin Neurobiol.

[7] Jones SA, Shim SH, He J, Zhuang X (2011). Fast, three-dimensional super-resolution imaging of live cells.. Nat Methods.

[8] Heilemann M, van de Linde S, Wolter S, Sauer M (2010). The effect of photoswitching kinetics and labeling densities on super-resolution fluorescence imaging.. Journal of Biotechnology.

[9] Sengupta P, Jovanovic-Talisman T, Skoko D, Renz M, Veatch SL (2011). Probing protein heterogeneity in the plasma membrane using PALM and pair correlation analysis.. Nat Methods.

[10] Kiskowski MA, Hancock JF, Kenworthy AK (2009). On the use of Ripley's K-function and its derivatives to analyze domain size.. Biophys J.

[11] Kolin DL, Wiseman PW (2007). Advances in image correlation spectroscopy: measuring number densities, aggregation states, and dynamics of fluorescently labeled macromolecules in cells.. Cell Biochem Biophys.

[12] Radenovic A, Annibale P, Scarselli M, Kodiyan A (2010). Photoactivatable Fluorescent Protein mEos2 Displays Repeated Photoactivation after a Long-Lived Dark State in the Red Photoconverted Form.. Journal of Physical Chemistry Letters.

[13] Heilemann M, Endesfelder U, Malkusch S, Flottmann B, Mondry J (2011). Chemically Induced Photoswitching of Fluorescent Probes-A General Concept for Super-Resolution Microscopy.. Molecules.

[14] Tanaka KA, Suzuki KG, Shirai YM, Shibutani ST, Miyahara MS (2010). Membrane molecules mobile even after chemical fixation.. Nat Methods.

[15] Engelhardt J, Keller J, Hoyer P, Reuss M, Staudt T (2011). Molecular Orientation Affects Localization Accuracy in Superresolution Far-Field Fluorescence Microscopy.. Nano Letters.

[16] Sethna JP (2006). Statistical mechanics: entropy, order parameters, and complexity.

[17] Honerkamp-Smith AR, Veatch SL, Keller SL (2009). An introduction to critical points for biophysicists; observations of compositional heterogeneity in lipid membranes.. Biochim Biophys Acta.

[18] Gompper G, Schick M (1990). Lattice model of microemulsions.. Phys Rev B Condens Matter.

[19] Onsager L (1944). Crystal statistics I A two-dimensional model with an order-disorder transition.. Physical Review.

[20] Veatch SL, Cicuta P, Sengupta P, Honerkamp-Smith A, Holowka D (2008). Critical fluctuations in plasma membrane vesicles.. ACS Chem Biol.

[21] Brewster R, Pincus PA, Safran SA (2009). Hybrid lipids as a biological surface-active component.. Biophys J.

[22] Erickson J, Goldstein B, Holowka D, Baird B (1987). The effect of receptor density on the forward rate constant for binding of ligands to cell surface receptors.. Biophys J.

[23] Mendoza G, Metzger H (1976). Distribution and valency of receptor for IgE on rodent mast cells and related tumour cells.. Nature.

[24] Machta BB, Papanikolaou S, Sethna JP, Veatch SL (2011). Minimal model of plasma membrane heterogeneity requires coupling cortical actin to criticality.. Biophys J.

[25] Wilson BS, Steinberg SL, Liederman K, Pfeiffer JR, Surviladze Z (2004). Markers for detergent-resistant lipid rafts occupy distinct and dynamic domains in native membranes.. Mol Biol Cell.

[26] Wilson BS, Pfeiffer JR, Surviladze Z, Gaudet EA, Oliver JM (2001). High resolution mapping of mast cell membranes reveals primary and secondary domains of Fc(epsilon)RI and LAT.. J Cell Biol.

[27] Wilson BS, Pfeiffer JR, Oliver JM (2000). Observing FcepsilonRI signaling from the inside of the mast cell membrane.. J Cell Biol.

[28] Singer SJ, Nicolson GL (1972). The fluid mosaic model of the structure of cell membranes.. Science.

[29] Sil D, Lee JB, Luo D, Holowka D, Baird B (2007). Trivalent ligands with rigid DNA spacers reveal structural requirements for IgE receptor signaling in RBL mast cells.. ACS Chem Biol.

[30] Gosse JA, Wagenknecht-Wiesner A, Holowka D, Baird B (2005). Transmembrane sequences are determinants of immunoreceptor signaling.. J Immunol.

[31] Larson DR, Gosse JA, Holowka DA, Baird BA, Webb WW (2005). Temporally resolved interactions between antigen-stimulated IgE receptors and Lyn kinase on living cells.. J Cell Biol.

[32] Baddeley D, Crossman D, Rossberger S, Cheyne JE, Montgomery JM (2011). 4D Super-Resolution Microscopy with Conventional Fluorophores and Single Wavelength Excitation in Optically Thick Cells and Tissues.. PLoS ONE.

[33] Wu M, Holowka D, Craighead HG, Baird B (2004). Visualization of plasma membrane compartmentalization with patterned lipid bilayers.. Proc Natl Acad Sci U S A.

[34] Hancock JF, Prior IA (2005). Electron microscopic imaging of Ras signaling domains.. Methods.

